# Intranasal Oxytocin in Pediatric Populations: Exploring the Potential for Reducing Irritability and Modulating Neural Responses: A Mini Review

**DOI:** 10.20900/jpbs.20230008

**Published:** 2023-08-31

**Authors:** Kennet Sorenson, Emilee Kendall, Hannah Grell, Minjoo Kang, Christopher Shaffer, Soonjo Hwang

**Affiliations:** 1 Department of Psychiatry, University of Nebraska Medical Center, Omaha, NE 68198, USA; 2 Department of Pharmacy Practice, University of Nebraska Medical Center, Omaha, NE 68198, USA

**Keywords:** intranasal oxytocin, OXT pediatric, autism spectrum disorder (ASD), Prader-Willi (PWS), Phelan-McDermid (PMS), irritability

## Abstract

Endogenous neuropeptide Oxytocin (OXT) plays a crucial role in modulating pro-social behavior and the neural response to social/emotional stimuli. Intranasal administration is the most common method of delivering OXT. Intranasal OXT has been implemented in clinical studies of various psychiatric disorders with mixed results, mainly related to lack of solid pharmacodynamics and pharmacokinetics model. Due to intranasal OXT’s mechanism of reducing the activation of neural areas implicated in emotional responding and emotion regulation, a psychopathology with this target mechanism could be potentially excellent candidate for future clinical trial. In this regard, irritability in youth may be a very promising target for clinical studies of intranasal OXT. Here we provide a mini-review of fifteen randomized controlled trials in pediatric patients with diagnoses of autism spectrum disorder (ASD), Prader-Willi syndrome (PWS), or Phelan-McDermid syndrome (PMS). Most studies had small sample sizes and varying dosages, with changes in irritability, mainly as adverse events (AEs). Neuroimaging results showed modulation of the reward processing system and the neural areas implicated in social-emotional information processing by intranasal OXT administration. Further research is needed to determine the most effective dose and duration of OXT treatment, carefully select target psychopathologies, verify target engagement, and measure adverse event profiles.

## INTRODUCTION

Oxytocin, especially intranasal administration, has been used for clinical trials for various psychiatric diagnoses in the past. In this mini review, we aim to summarize recent clinical trials of intranasal oxytocin, especially for pediatric populations and propose future directions. Specifically, we focus on the importance of establishing pharmacodynamics and pharmacokinetics models of intranasal oxytocin in clinical population, and also select a candidate psychopathology for future clinical trials, preferably dimensional psychopathology based on neurobiological mechanism that can be the target mechanism of intranasal oxytocin; i.e., irritability in youths.

Oxytocin (OXT) is a hypothalamic neuropeptide that plays an important role in mammalian social processes [1], such as maternal nurturing, bonding, and social recognition. Beyond its social effects, OXT also regulates uterine contractions during childbirth and lactation [2]. It is widely believed that OXT exerts its behavioral and emotional effects via neural areas such as amygdala, whose role in the regulation of emotional and social behavior is well-established [3]. This is supported by the amygdala’s high density of OXT receptors [4]. Other brain areas that house OXT receptors include the hypothalamus, hippocampus, nucleus accumbens, insula, and striatum [5]. As for the mechanism of action, previous imaging studies have demonstrated that intranasal OXT reduces amygdala responses to threatening cue/negative emotional stimuli [6]. Intranasal OXT administration has been repeatedly shown to induce increases in neural activity in the anterior cingulate cortex and middle prefrontal cortex (mPFC) as well [7]. On the other hand, there are also other studies showing that OXT can enhance the activation of the amygdala, [8–10], nucleus accumbens, striatum and certain cortices and gyri in the frontal lobe [11]. The direction of changes in the neural areas mainly depended on the target population, as well as the methods of measurement (specifically, implementation of various types of cognitive-affective tasks).

The most commonly used mode of OXT administration is intranasal [12], given the size of the neuropeptide (1007.2 g·mol^−1^) [13] in comparison to, for example, dopamine (153.18 g·mol^−1^) [14]. The olfactory receptor neurons of olfactory epithelium contains cilia that can deliver intranasal OXT to the olfactory bulb at the base of human brain [5]. In the olfactory bulb, OXT can bind to its neural receptors and from there can be transported to the other areas of the central nervous system (CNS), including the limbic system and hypothalamus [15]. OXT is metabolized by aminopeptidase, and specifically in the CNS by insulin-regulated aminopeptidase [16,17]. The average half-life of OXT is relatively short. In adult men, 26 IU of intranasal OXT resulted in substantially increased blood OXT concentrations at 30 min post-administration and returned to near baseline by the 90-min mark for most participants, though several still had elevated levels 150 min after administration. [18]. The half-life of OXT in blood is typically less than 2 min. However, in cerebrospinal fluid, its half-life can be as long as 28 min [5].

There are many previous studies including clinical trials of intranasal OXT for various psychiatric diagnoses. Major psychiatric diagnoses used for OXT clinical trials include autism spectrum disorder (ASD), borderline personality disorder (BPD), major depressive disorder and schizophrenia [12,19,20]. Overall, findings have been mixed, with some reporting a mild reduction in symptoms [21–23] and others reporting no significant change. [24–26]. However, neuroimaging studies (primarily with fMRI) have consistently revealed promising findings following OXT administration [7]. A systematic review and meta-analysis (Grace et al. [7]) found consistent increases in left superior temporal gyrus activity following intranasal OXT administration across 39 fMRI studies. The left superior temporal gyrus is associated with auditory language processing and social cognition, and its dysfunction has been implicated as a potential etiology of autism spectrum disorder [27].

These previous findings lead to the possibility of designing clinical trials by selecting a trans-diagnostic psychopathology that potentially has a neurobiological mechanism that can be targeted by intranasal OXT (in this case, hyperactivation of emotion responding areas to negative emotional cue). There are a few potential benefits of focusing on trans-diagnostic psychopathology as a candidate for OXT clinical trials.

First, the complexity of psychiatric disorders, including complex clinical presentation of multiple psychopathologies, heterogeneity of symptom profiles at individual level, and frequent presence of comorbid disorders [28] really limits the utility of clinical trials focusing on categorical psychiatric diagnoses. Often, the efficacy of clinical trials fail to be translated into the effectiveness of the treatment agent in real world setting due to these reasons [29].

**Figure 1.**
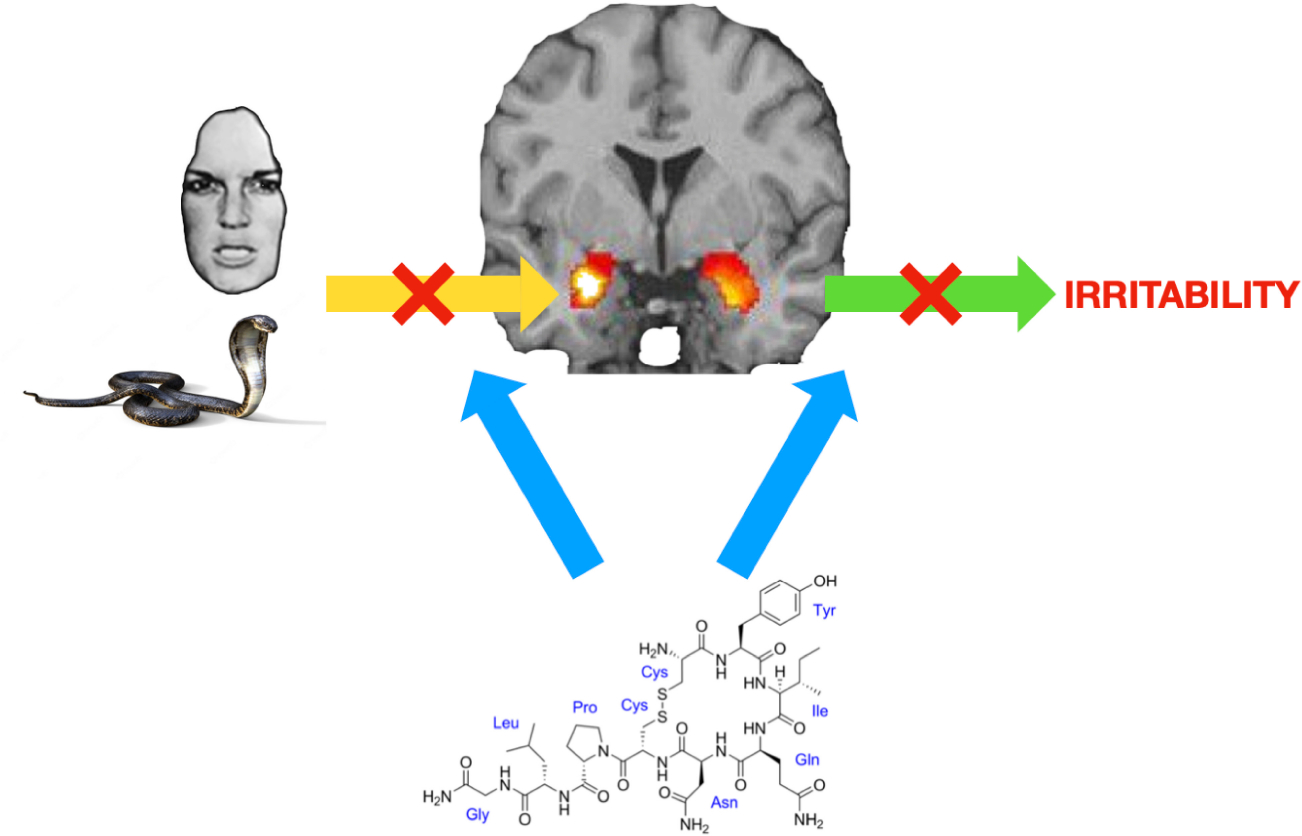
The potential modulatory neural impact of the neuropeptide hormone oxytocin as intranasal administration on the core mechanism of irritability. Oxytocin is known to reduce activation of the acute threat response system (in this figure, amygdala) to emotional stimuli (→). The most established neurobiological mechanism of irritability is increased activation of the amygdala in the acute threat response system to emotional stimuli (→), resulting in irritability (→).

Second, selecting patients for clinical trial becomes exhaustive and expensive process, which often creates the issue of type II error due to small sample sizes [30]. Lastly without consideration of underlying neurobiological mechanism, it is hard to interpret the symptom profile changes by any given clinical trial to guide future direction of clinical practice and clinical/research studies [31]. In this regard, as a target psychopathology of intranasal OXT clinical trials, ***irritability*** might be an excellent candidate for various reasons. Irritability, especially in the pediatric population, seems to share common neurobiological mechanisms, particularly in the context of disruptive mood and behavior disorders [32,33]. In this regard, many previous studies demonstrated abnormalities in both threat and reward processing in children and adolescents who display significant levels of irritability [32,34,35]. Consistently, studies have shown a tendency toward hyper-reactivity in the neural areas implicated in processing threat cues or negative emotional stimuli in irritable children, mainly mediated by the amygdala (Figure 1) and ventromedial prefrontal cortex [32]. In various populations with different psychiatric diagnoses, when a neuroimaging modality is applied, a pattern of intranasal OXT reducing the activation of these areas in response to negative emotional stimuli is often observed [7,36–38]. Taken together, this would indicate the potential role of intranasal OXT in ***target engagement*** of neurobiological mechanisms of irritability [39], particularly in youths with diagnoses of disruptive behavior and mood disorders [33]. Furthermore, several functional connections between regions of the brain have been connected to irritability, including increased connectivity between the anterior cingulate cortex and the rostral medial prefrontal cortex [40], as well as decreased connectivity between the amygdala and both the medial prefrontal cortex [32] and the precuneus [41].

Currently, there is no study specifically focusing on irritability in youth as the target psychopathology of an intranasal OXT clinical trial. However, at present, there are still many previous studies that measured irritability as a secondary outcome, especially in the youth with Autism Spectrum Disorder (ASD), and conditions associated with ASD like Prader-Willi syndrome (PWS) [42,43] and Phelan-McDermid syndrome (PMS) [44].

Irritability is a major manifestation of ASD, occurring in an estimated 43% of pediatric ASD patients, including those classified as high-functioning [45]. Similarly, irritability has also been found to be a clinical phenotype of youths with PWS [46] and PMS [47]. Autism spectrum disorder (ASD) is characterized by the DSM-5 as a neurodevelopmental disorder marked by impaired social abilities and restricted and repetitive behaviors [31]. Due to OXT’s role in social information processing [48], it has become an appealing candidate for clinical trials for youth with ASD, with limited social abilities as the primary outcome measurement. Studies have found connections between OXT receptor (OXTR) polymorphism and impaired social functioning [49–51], which is a hallmark feature of ASD and suggests that OXTR dysfunction may contribute to the ASD phenotype. In addition to social deficits, youth with ASD also have high prevalence of irritability and emotional dysregulation [45,52]. Mikita et al. [53] found that in boys aged 10–16, those with high-functioning ASD were recorded to have significantly higher levels of cortisol and greater heart rate variability during stressful situations compared to control, suggesting that irritability could be considered as a measure of symptom severity in ASD patients. Proposed reasons for this symptom include frustration stemming from difficulty communicating thoughts, interruption of repetitive behaviors and exposure to adverse sensory experiences [45].

Though a distinct phenotype from ASD, PWS shares clinical characteristics with autism, including irritability [54]. PWS is caused by deficient expression of genes from paternal chromosome 15q11-q13 [55]. Common symptoms of PWS include cognitive, endocrine and behavioral dysfunction, including irritability, which often manifests itself as temper tantrums [55]. Rice et al. [56] found that, in patients with the 15q11-q13 PWS subtype, salivary OXT levels were inversely correlated with symptom severity, suggesting that OXT deficiency may play a role in the PWS symptomatology. Further studies have connected OXT deficiency with aberrant behaviors observed in patients with PWS [57]. Again, PWS’s behavioral phenotype has made it an attractive disorder for OXT research.

Phelan-McDermid syndrome (PMS) is a disorder that often presents with clinical features of ASD, and it occurs due to a 22q13.3 chromosomal deletion affecting the *SHANK3* gene [58]. The *SHANK3* gene is expressed abundantly in the brain and is important in establishing connections between neurons [59]. Disruptions in these neural links is believed to underlie the behavioral symptoms of both ASD and PMS [59]. In rats with *Shank3* mutations displaying impaired social recognition and decreased synaptic plasticity, treatment with OXT improved social cognition and memory, suggesting that OXT administration may be able to attenuate some of the behavioral disturbances seen in PMS (and potentially ASD) [60]. PMS can manifest with both physical (e.g., large ears, dysplastic toenails, hypotonia) and psychiatric features (e.g., severe global developmental delay, language impairment, aggressive behavior) [58]. Like PWS, PMS has symptomatic overlap with ASD, including aggression/irritability [58].

Reactive Attachment Disorder (RAD) is a psychiatric disorder characterized by disturbed attachment patterns and difficulties forming healthy emotional connections with caregivers [61]. Because of OXT’s connection to social bonding [48], RAD has become an interesting target of intranasal OXT therapy as well.

Because of OXT’s connection to social functioning [48], ASD, PWS and PMS have been the foci of most intranasal OXT research to this point. While social functioning is not the focus of this review, all three of these disorders can manifest clinically with irritability [53,54,57]. In this mini review, we present the summary of results from 15 clinical trials published between 2014 and 2023 that explore the effects of intranasal OXT on pediatric patients with ASD, PWS, PMS or RAD.

## METHODS

“Intranasal Oxytocin pediatric randomized controlled trial irritability” was entered into the search bar of both PubMed and Cochrane Central Register of Controlled Trials. The search details were ((“pediatrics”[MeSH Terms] OR “pediatrics”[All Fields] OR “pediatric”[All Fields]) AND intranasal[All Fields] AND (“oxytocin”[MeSH Terms] OR “oxytocin”[All Fields]) AND (“irritable mood”[MeSH Terms] OR (“irritable”[All Fields] AND “mood”[All Fields]) OR “irritable mood”[All Fields] OR “irritability”[All Fields]) AND (“randomized controlled trial”[All Fields] OR “randomized controlled trials as topic”[MeSH Terms] OR “randomized controlled trial”[All Fields] OR “randomised controlled trial”[All Fields])) AND (“2013/01/01”[PubDate]: “2023/12/31”[PubDate]).

“Intranasal Oxytocin pediatric irritability” was also entered into the search bar of Embase and filters were applied to meet the requirements of this review. The search details were (**‘intranasal oxytocin’** OR (**intranasal** AND (**‘oxytocin’**/exp OR **oxytocin**))) AND ([newborn]/lim OR [infant]/lim OR [child]/lim OR [preschool]/lim OR [school]/lim OR [adolescent]/lim) AND [2013–2023]/py AND (**‘antisocial personality disorder’**/dm OR **‘anxiety disorder’**/dm OR **‘attention deficit hyperactivity disorder’**/dm OR **‘autism’**/dm OR **‘behavior disorder’**/dm OR **‘disruptive behavior’**/dm OR **‘hyperactivity’**/dm OR **‘mood disorder’**/dm OR **‘prader willi syndrome’**/dm OR **‘psychosocial disorder’**/dm) AND (**‘controlled study’**/de OR **‘double blind procedure’**/de OR **‘human’**/de OR **‘randomized controlled trial’**/de).

The Cochrane Risk of Bias Tool [62] was used to assess the quality of the studies gathered. The biases particularly targeted by these assessments were selection bias (comprised of random sequence generation and allocation concealment), reporting bias (selective reporting), performance bias (blinding—participants and personnel), detection bias (blinding—outcome assessment) and attrition bias (incomplete outcome data).

In order to be considered for inclusion, RCTs also needed to meet several criteria. They needed to be published in peer-reviewed journals in the last 10 years and to have examined the effects of intranasal OXT administration in pediatric patients (aged 6 months to 18 years) diagnosed with ASD, PWS or PMS. Overlapping diagnoses were not considered as exclusionary. Trials must also have included placebo groups and utilized at least one of several pre-determined measures of efficacy. These included the aberrant behavior checklist (ABC), the revised repetitive behavior scale (RBS-R), the social responsiveness scale (SRS), the strengths and difficulties questionnaire (SDQ), and brain imaging (fMRI and MEG). In general, the ABC measures psychiatric and behavioral abnormalities across several different domains, including irritability, lethargy/social withdrawal, stereotypic behavior, hyperactivity/noncompliance and inappropriate speech [63]. Because of its high reliability and validity, the ABC has been used frequently in both pediatric and adult psychiatric research [63]. The RBS-R is a relatively easy-to-complete parent-scored questionnaire that focuses specifically on repetitive behaviors, which are often associated with ASD [64]. The SRS has been used both clinically and in research to screen for and measure the symptoms of social impairment associated with ASD [65]. The SDQ is generally used to assess the behavioral and emotional characteristics of children. The SDQ measures several different domains of functioning, including peer relationships, hyperactivity, prosocial behavior, conduct problems (such as aggression) and emotional symptoms (including anxiety and distress) [66]. Because it measures both symptoms of aggression and anxiety, the SDQ may work well as a surrogate measure of irritability. No studies using direct measures of irritability (such as the Affective Reactivity Index [67] or the Brief Irritability Test [68] were found, so indirect measures of potential irritability (via hypothesized mechanisms of irritability development [45]) had to be used. Duration of administration (i.e., single dose vs chronic use) was not used as an exclusionary factor, nor was dosage of OXT administered. Ultimately, 15 RCTs met inclusion criteria.

## RESULTS

Initial screening resulted in forty-three potentially eligible articles. Of these, 15 met the full inclusion criteria, and their summaries are included in Table 1. Eleven of the fifteen articles included participants diagnosed with ASD [1,25,26,69–76], regardless of Phelan-McDermid syndrome (PMS) status. Two [42,43] of the fifteen articles included participants diagnosed with Prader-Willi syndrome (PWS). In both of these trials, participant ASD diagnosis was unknown [42,43]. Most of the studies had relatively small sample sizes (18 to 87), except one study by Sikich et al. [26] (*n* = 290). In one study, all participants were diagnosed with ASD, the etiology of which was PMS [44]. One of the fifteen trials examined intranasal OXT’s effect on patients with reactive attachment disorder (RAD) [77]. All trials included were double-blinded. Three of the fifteen trials [1,69,77] only administered single doses of intranasal OXT to participants. Of the trials administering multiple doses, Dadds et al. [25] was the shortest, only spanning five days. The other eleven trials all ran for at least one week, ranging from one to twenty-four weeks.

Each of the fifteen trials included administered OXT or a placebo intranasally, though daily dosages varied. Overall, the lowest dosage used by an included trial was 12 IU [1,25,72], while the highest target dosage was 48 IU [25,44]. Some trials adjusted dosages depending on several different factors. Fastman et al. [44] administered 48 IU daily to its first seven participants but then lowered it to 24 IU after witnessing increased irritability at the higher dose. Damen et al. [42] administered doses ranging from 16–40 IU daily and provided higher doses to participants with larger body surface areas. Gordon et al. [1] and Korisky et al. [73] stratified dosages based on the ages of their participants, while Dadds et al. [25] provided 24 IU daily to participants weighing more than 40 kg and 12 IU daily to participants weighing under 40 kg. Miller et al. [43] provided all participants with 16 IU daily. However, Sikich et al. [26] dosed participants differently. At the start of the trial, all participants were given 8 IU in the morning. By week 8, the goal dosage was 48 IU daily (given via 2 doses of 24 IU) [26]. Once a participant had been on 48 IU for 7 weeks, doses could be increased by 16 IU every 4 weeks (with a cap at 80 IU), be reduced by 8 to 16 IU or be held at 48 IU [26]. Six trials [69,70,71,74,76,77] administered 24 IU to every participant in the treatment group. No relationship between the dosages of intranasal OXT administered and the clinical outcome were found across the trials examined in this review.

To merit inclusion in this review, studies needed to measure effects using at least one of a predetermined set of means. Three of the studies [26,43,44] employed the Aberrant Behavior Checklist modified Social Withdrawal subscale (ABC-mSW), and one [74] used the Aberrant Behavior Checklist—Parent subscale. Of the four trials using this scale, only one study (Miller et al.) [43] showed consistently improved ABC scores throughout the trial, suggesting limited efficacy of OXT in promoting prosocial behaviors.

The revised repetitive behavior scale (RBS-R) was used by seven studies [42–44,70,72,74,75]. Of the seven studies employing the RBS-R, only two [43,75] found that intranasal OXT reduced the prevalence of repetitive behaviors, suggesting limited efficacy in addressing this symptom. Additionally, another study (Le et al.) [75] also reported that eye-tracking data revealed that the OXT group spent more time viewing dynamic social stimuli than geometric stimuli. Conversely, five studies (Damen et al. [42], Fastman et al. [44], Yatawara et al. [70], Daniels et al. [72] and Guastella et al. [74]) did not observe significant improvements in RBS-R scores between the OXT and placebo groups.

The social responsiveness scale (SRS) was used by eight of the fifteen studies [25,26,42,70,71,72,74,75], five of which [25,42,70,71,75] found that OXT improved the SRS scores of participants, suggesting that intranasal OXT may improve the social functioning of pediatric patients with ASD or PWS. One study (Daniels et al. [73]) reported no significant differences were observed in SRS-2 or RBS-R between the groups receiving OXT and placebo during double-blind procedures. However, improvement was noted during the single-blind phase of the trial in the SRS-2. Another study (Guastella et al. [74]) discovered that there were no significant differences in scores on the Clinical Global Impressions (CGI) scale and Social Responsiveness Scale (SRS-2) between the OXT and placebo groups in the older age group (6–12). In the younger age group (3–5), there was no significant effect on SRS-2 scores, but the younger treatment group showed greater improvements in CGI scores compared to the older age group. Secondary measures were not significantly affected by OXT administration. Le et al. [75] found that treatment with OXT resulted in significant improvements in the Autism Diagnostic Observation Schedule (ADOS-2) and SRS-2 scores compared to placebo. The OXT group also showed greater improvement over placebo in the Adaptive Behavior Assessment System-II (ABAS-II) and Repetitive Behavior Scale-Revised (RBS-R) scores. Dadds et al. [25] did not find significant improvements in emotion recognition, SRS scores, social interaction skills, or general behavioral adjustment with OXT, and Sikich et al. [26] did not see significant improvements in SRS-2 scores either. In contrast, Le et al. [75] found that treatment with OXT resulted in significant improvements in SRS-2 scores compared to placebo, as did Damen et al. [42], Yatawara et al. [70] and Parker et al. [71].

The strengths and difficulties questionnaire (SDQ) was used by one study [76]. Karbasi et al. [76] found that OXT administration led to significant improvements in teacher and parent SDQ scores, as well as GARS-2 (Gilliam Autism Rating Scale-Second Edition) scores, suggesting a therapeutic effect on behavioral difficulties.

Four studies [1,69,73,77] used brain imaging (fMRI [1,69,77] and MEG [73]) to examine the effects of intranasal OXT administration on pediatric ASD patients. In these, intranasal OXT was found to increase activation of reward processing brain systems in response to nonsocial stimuli [69] and enhance activity and connectivity in brain regions involved in perceiving social-emotional information during social stimuli [1,73]. Gordon et al. [1] found that the administration of OXT led to increased activity in brain regions crucial for processing social-emotional information and enhanced the connectivity between nodes of the brain’s reward and social-emotional processing systems specifically during social stimuli, including increased functional connectivity of the amygdala with the ventromedial PFC, the orbitofrontal cortex and the precuneus. Similarly, Korisky et al. [73] found that OXT increased neural activity in the frontal regions of the brain in response to social stimuli, as well as in the left hemisphere regardless of the stimulus. Takiguchi et al. [77] discovered that OXT administration enhanced activation in the right middle frontal gyrus and striatum, consistent with enhancements in the mesolimbic dopaminergic reward system. Reduced activation was observed in the right precentral gyrus during a reward task. Greene et al. [69] found that OXT increased activation of brain systems processing rewards, especially during nonsocial incentive salience stimuli. Additionally, Greene et al. [69] found decreased connectivity between the left anterior cingulate cortex and the right superior and left medial frontal gyri. These findings collectively indicate that intranasal OXT administration modulates brain activity and connectivity in regions associated with reward processing, social-emotional information processing, stress regulation, and motivation, thus highlighting the potential therapeutic implications of OXT in addressing social and emotional difficulties in individuals with ASD.

Three [43,44,70] of the fifteen studies reported adverse reactions potentially attributed to intranasal OXT use. Yatawara et al. [70] stated that participants in the OXT-treatment group reported more frequent urination, thirst and constipation, relative to controls. Miller et al. [43] reported that the OXT-treatment group experienced increased intranasal and psychological irritation. Fastman et al. [44] found that, at 48 IU daily, participants experienced greater psychological irritability. Subsequent participants were dosed at 24 IU daily, and this effect ceased.

In summary, there is still lack of consistent findings on the clinical efficacy of intranasal OXT, which is highly dependent on the target population, selection of primary/secondary outcomes, and clinical study/trial design. Many studies implementing measurement of biological changes (including neuroimaging) showed more consistent findings in the areas implicated in social and/or emotional information processing.

## DISCUSSION

The studies included in this review yielded mixed results. First of all, most pediatric intranasal OXT trials did not include scales to measure irritability directly in favor of focusing on the social dysfunction and repetitive behaviors associated with ASD, PWS and PMS. There is no clinical trial of intranasal OXT specifically targeting irritability as the primary outcome in pediatric population so far. As for the primary outcomes measured, the clinical efficacy of intranasal OXT was still inconclusive. FMRI data were inconsistent across the three trials that implemented it [1,69,77]. Two [1,69] of these trials were single-dose administration studies that found that intranasal OXT administration activated the mesocorticolimbic system, which is involved in reward processing [78]. Both the lateral septum (LS) and the bed nucleus of the stria terminalis (BNST) are believed to be involved in both the brain’s social reward network and its mesolimbic reward system [78] and are known to house OXT receptors [78]. Thus, increased activity in mesocorticolimbic system after OXT administration compared to placebo is a promising finding, as it may indicate increased social processing in ASD patients. Additionally, these two trials [1,69] found changes in connectivity that are implicated in reducing irritability [40,41,69]. However, these changes in connectivity were not consistent between the two trials and were not also observed in the other two imaging studies included in this review [73,77]. Furthermore, none of these studies implemented fMRI tasks of emotional responding/emotion processing paradigm, which could be potentially helpful to verify the target engagement of intranasal OXT, given the previous studies showing this effect [7]. Instead, these studies utilized tasks focused on social perception [1], social [69] and non-social [69,77] incentives and identification of social and non-social stimuli [73]. Though increased activity in areas such as the ventral striatum [1,69,77] (associated with reward processing and motivation and thought to mediate interaction between the prefrontal cortex and amygdala [79]), anterior cingulate cortex [69,73] (associated with motivation, decision making, learning and social processing [80]), and dorsolateral prefrontal cortex [69,73] (associated with executive control functions [81]) were observed in multiple different studies, they were observed in different contexts (i.e., during different tasks) and were not consistent across all trials. Further imaging research examining the effects of intranasal OXT on activity of and functional connectivity between the amygdala, prefrontal cortex and associated brain regions in response to threat cue is warranted in pediatric patient populations to further elucidate these relationships.

In addition to this, scores of the ABC and RBS-R were less consistent across the studies. Of the three studies utilizing the ABC, only one [43] saw significant score improvements between placebo and treatment groups. This study [43] also happened to be the shortest and lowest-dosing of the three trials, with only one 16 IU dose administered over the course of five days. Fastman et al. [44], Sikich et al. [26] and Guastella et al. [74] lasted 12 weeks, 24 weeks and 12 weeks, respectively, administered doses ranging from 24–48 IU daily, and none found significant changes in ABC scores.

Similarly, Miller et al. [43] and Le et al. [75] were the only trials to see RBS-R scores improve, with the other five trials using the RBS-R [42,44,70,72,74], all failing to observe improved scores in their treatment groups.

SRS scores showed heterogeneity across the trials as well, with five of the eight studies showing improved SRS scores [25,42,70,71,75]. Although Sikich et al. [26] showed the most rigorous method with a relatively large sample size (*n* = 290), it could be possible that follow-up studies with different outcome measures in different population may show different results. In this regard, again, measurement of target engagement would be critical.

There were two studies reporting irritability, as potential AEs of intranasal OXT (especially one at 48 IU, which improved with the decreased dose of 24 IU). There are other previous studies reporting increased irritability and/or aggressive behavior as adverse events (AEs) of intranasal OXT in youth with ASD (AEs) [73]. Irritability/emotional dysregulation in ASD is a very prominent clinical issue. There are hypotheses about its etiology [49], but so far little has been concluded about its neurobiological mechanism [82,83]. It is possible that the impact of intranasal OXT on children with ASD might be significantly different than neurotypical youths. As such, the impact of OXT may differ depending on the target population and the type of dependent measure used to index irritability and aggression. Further research using more direct measures of irritability are needed to confirm this relationship.

There are several crucial issues impeding advancement in this area. One is that intranasal OXT pharmacokinetics are not fully understood. It is not known how much OXT reaches target sites in the brain via intranasal administration. Neumann et al. [84] demonstrated that intranasal OXT administration led to increased plasma and brain extracellular fluid concentrations of OXT in rodents. It is encouraging that recently there are primate [85,86,87] and human [88] studies that have shown increased level of CSF OXT as well as neural level change as a function of different doses after intranasal administration. Advancement of technology in detecting OXT level as well as implementing neuroimaging (fMRI) enhanced the scientific rigor of measuring intranasal OXT activities in these studies [33,39]. Intranasal OXT pharmacokinetics and the ability of intranasal OXT to reach its target sites in the central nervous system are still matters of ongoing research [89].

Additionally, much higher concentrations of OXT may be needed to influence behavior compared to peripheral functions of OXT [31]. In Mens et al. [90], subcutaneous OXT injections into rats resulted in up to 500-fold increases in plasma concentrations of OXT and moderate increases in CSF concentrations (40 pg/mL to 150 pg/mL). However, only 0.002% of the injected OXT reached the central nervous system after 10 min at maximal CSF concentrations [91]. To determine this, it would be critical to consider various factors that can contribute to the results, including the mode of delivery (most studies implemented liquid formula, but there are other studies used different methodologies, the target population (healthy vs. patients with various psychiatric diagnoses), and the methodology to measure the end-point behavioral and psychological effects [89,91–93]^.^ For example, there is very little to no data on the relation between induced neural change by intranasal OXT and end-point behavioral/psychological changes. Further research is warranted to establish these relations. Future studies should provide more information on which dosages and administration schedules yield the greatest clinical benefit.

As both irritability and aggression (especially reactive aggression) occur in response to negative emotional stimuli/threat cues (Figure 1) [94], and the amygdala is one of the major areas implicated in negative emotional stimuli/threat cue processing, the role of intranasal OXT for aggression in relation to irritability is worth further investigation. Similar to the neuroimaging findings [8,9,10,36–38,92], previous studies have shown various results. Berends et al. [95,96] found that lower urine OXT levels were correlated with more aggressive behavior [95], and that intranasal OXT administration reduced aggressive behaviors in young adult males [96]. However, other studies have found that intranasal OXT administration increases aggressive behavior in healthy adults [97,98]. Again, selection of a specific target population, reliable measurement of the end point behavioral impact (in this case, aggression and especially reactive aggression), and establishing dose-response relationship would be critical.

Our initial aim was to have thorough investigation of the role of intranasal OXT on pediatric population. However, there has been no previous study specifically aimed to address this question. Only two recent studies of intranasal OXT administration measured irritability as adverse events [43,44]. Currently, our group is in the process of publishing our clinical trial results of specifically addressing this question (irritability as the primary outcome for a randomized double blind clinical trial of intranasal OXT for adolescents with high levels of irritability). In the future clinical trials, verification of target engagement would be critical; i.e., decrease in the emotion regulation areas by intranasal oxytocin to negative emotional stimuli, which has been repeatedly shown in adult population [7,99,100,101].

In addition, it would be critical to implement standardized and comprehensive evaluation of irritability in the clinical trials. None of the trials included measured irritability directly using scales such as the Affective Reactivity Index (ARI) or the Brief Irritability Test (BITe), but all used scales that measured potential underlying etiologies [45] of irritability. Because there is still much unknown about the causes of irritability in ASD, PWS and PMS, the use of more specific irritability scales is needed to elucidate the relationship between intranasal OXT and irritability in these patients. Two studies [43,44] reported increased psychiatric irritability as a side effect of OXT administration. However, neither of the studies reported increased irritability via any standardized method or scale, direct or indirect. Miller et al. [43] saw improved RBS-R and ABC scores in the treatment group, despite also observing increased irritability, while Fastman et al. [44] observed no significant difference in the ABC and RBS-R scores between groups. Nonetheless, iatrogenic irritability is something that should be followed more closely through scales such as the BITe or ARI as further research on intranasal OXT is done, in order to monitor the relationship between irritability and intranasal OXT.

## CONCLUSIONS

Despite decades of research, many unknowns still exist about the therapeutic efficacy of intranasal OXT. Though clinical trials have yielded inconsistent behavioral effects thus far, fMRI studies have shown more consistent, although still inconclusive, findings of intranasal OXT administration on neural activity. At present, most pediatric intranasal OXT trials have neglected to measure the effect of OXT on irritability. Thus, we advocate for a broader program of research within pediatric populations that considers specific trans-diagnostic targets, such as irritability. Such a program would allow researchers to engage in more targeted and nuanced exploration of the efficacy of intranasal OXT and its impact on pro-social behavior, neural responses, and emotional regulation across a range of psychiatric disorders. Additionally, such a trans-diagnostic approach could promote the identification of commonalities and shared pathways, paving the way for more efficient and effective treatments in clinical trials. Furthermore, we propose a few next steps to achieve sufficient scientific rigor. First, establishing pharmacodynamics/pharmacokinetics model would be critical. Although there are studies on the effects of intranasal OXT as a function of dose (e.g., Quintana et al. [91] and Spengler et al. [102]), there is still no established comprehensive pharmacodynamics/pharmacokinetics model of intranasal OXT. To establish this, careful consideration of mode of administration, target population, and methodology to measure end-point behavioral changes would be critical. Second, target engagement and selection of psychopathology that could be targeted by intranasal OXT should be carefully considered. It would be critical to implement method(s) to verify target engagement, in addition to symptom profile changes. Targeting a psychopathology that has shown neurobiological mechanism that can be impacted by intranasal OXT would be critical. We propose increased activation of emotional responding/emotion regulation area and related psychopathology (for example, irritability) would be very promising. Adverse event profiles (AEs) should be measured in structured way as well.

## Figures and Tables

**Table 1.** Randomized clinical trials of intranasal OXT for youth with ASD in the last 10 years.

Study	Study size	Diagnosis	Diagnostic criteria	Administration	Dosage	Duration	Outcomes	Results	Side effects
Greene, 2018 [69]	28	Autism spectrum disorder (ASD)	Children/Adolescents diagnosed with ASD without comorbidity.	Intranasal	24 IU or Placebo	2 days	2 fMRI scans while completing social and nonsocial incentive delay tasks	Intranasal OXT administration increased activation of mesocorticolimbic brain systems that process rewards in ASD; primarily observable during the processing of nonsocial incentive salience stimuli. Decreased connectivity between the anterior cingulate cortex and frontal regions, including the rostral medial PFC.	None reported.
Sikich, 2021 [26]	290	Autism spectrum disorder (ASD)	Children/Adolescents aged 317 who met DSM-5 criteria for ASD.	Intranasal	48 IU daily or Placebo	24 weeks	Least-squares mean change from baseline on the Aberrant Behavior Checklist modified Social Withdrawal subscale (ABC-mSW); Sociability Factor score, SRS-2, ADOS-2 comparison score, VABS-II socialization standard score, SB5 abbreviated IQ	ABC-mSW change from baseline was not significantly different between OXT treatment group and placebo group; secondary outcomes were not significantly different between groups.	No significant difference between treatment groups.
Yatawara, 2016 [70]	31	Autism spectrum disorder (ASD)	Children between ages of 3–8 who met DSM-IV-TR diagnostic criteria for Autistic Disorder, Asperger’s Disorder or Pervasive Developmental Disorder-Not Otherwise Specified (PDD-NOS).	Intranasal	24 IU daily or Placebo	5 weeks	SRS-P, ADOS, DBC-P, RBS-R-P, CSQ	Significant improvements in SRS-P and DBC-P for the oxytocin condition; no significant difference in ADOS, RBS-R-P and CSQ between oxytocin and placebo groups.	Minimal; occasional thirst, frequent urination and constipation
Dadds, 2014 [25]	38	Autism spectrum disorder (ASD)	Male youths (7–16 years old) who met DSM-IV-TR criteria for Autistic disorder, Asperger’s disorder or PDD-NOS.	Intranasal	12 IU daily or 24 IU daily or Placebo	5 days	DISCAP axis 1 severity, CARS total impairment scale, OSU autism global impression scale, Facial emotion recognition task, Video observation of social interaction skills, Video observation of repetitive behaviors, SRS autistic mannerisms	Oxytocin did not significantly improve emotion recognition, social interaction skills, or general behavioral adjustment	No significant difference between treatment groups.
Parker, 2017 [71]	32	Autism spectrum disorder (ASD)	Children (6–12 years old) who met DSM-IV criteria for Autistic disorder, Asperger’s disorder or PDD-NOS or DSM-V criteria for Autism spectrum disorder.	Intranasal	24 IU daily or Placebo	4 weeks	SRS, pre- and post-treatment plasma OT concentration, DOTES	Significant SRS improvement in OXT-treatment group; lowest pretreatment OXT concentrations showed the greatest social improvement; no observed decrease in repetitive behaviors or anxiety.	No significant difference between treatment groups.
Gordon, 2016 [1]	21	Autism spectrum disorder (ASD)	Children aged 10–18 years with diagnosis of ASD.	Intranasal	24 IU, 18 IU, 12 IU or Placebo	2 days	fMRI	OXT administration increased activity in brain regions important for perceiving social-emotional information; OXT administration also enhances connectivity between nodes of the brain’s reward and social-emotional processing systems during social stimuli. OXT administration led to increased connectivity between the anterior and posterior precuneus and the nucleus accumbens and amygdala; increased connectivity between ventromedial PFC and orbitofrontal cortex with nucleus accumbens and amygdala.	None reported.
Miller, 2017 [43]	24	Prader-Willi syndrome (PWS)	Children aged 5–11 diagnosed with PWS.	Intranasal	16 IU daily on days 2–6 or Placebo	2 weeks	RBS-R, ABC, HQ, CGI	Improvement in RBS-R, ABC, HQ, and CGI scores	Nasal irritation and irritability.
Damen, 2021 [42]	26	Prader-Willi syndrome (PWS)	Children aged 3–11 diagnosed with PWS.	Intranasal	16–40 IU daily or Placebo	3 months	Oxytocin Questionnaire, Dykens hyperphagia questionnaire, RBS-R, SRS-P	Oxytocin Questionnaire scores improved in the OXT group, as did SRS-P scores, but social behavior, hyperphagia and RBS-R scores remained largely unchanged.	None reported.
Fastman, 2021 [44]	18	Phelan-McDermid Syndrome (PMS)	Children between the ages of 5–17 years with pathogenic deletions or variations of the *SHANK3* gene.	Intranasal	48 IU daily (first seven participants) and 24 IU daily or Placebo	12 weeks	ABC-mSW, RBS-R, CGI-I, SSP, Vineland-II, MSEL, MCDI	No significant improvement on any of the measures between OXT and placebo groups.	Increased irritability at 48 IU daily.
Daniels, 2023 [72]	77	Autism spectrum disorder (ASD)	Children between the ages of 8–12 with a formal diagnosis of ASD.	Intranasal	12 IU daily or Placebo	4 weeks	SRS-2; secondary measures included RBS-R, SCARED parent, SCARED child, ASCQ anxious, ASCQ avoidant, ASCQ secure	No significant improvement on any of the measures between OXT and placebo groups in double-blind procedures. Improvement noted during single-blind phase of trial.	None reported.
Korisky, 2022 [73]	25	Autism spectrum disorder (ASD)	Adolescent males aged 12–18.	Intranasal	24 IU daily for participants aged 13–18, 16 IU daily for participants aged 12 or Placebo	1 week	Magnetoencephalography (MEG)	OXT increased neural activity in the frontal regions in response to social stimuli and in the left hemisphere regardless of the stimulus.	None reported.
Guastella, 2023 [74]	87	Autism spectrum disorder (ASD)	Children between the ages of 3–12 years old with a formal diagnosis of ASD.	Intranasal	32 IU daily (16 IU in the morning, 16 IU at night) or Placebo	12 weeks	SRS-2, CGI; secondary measures included RBS-R, ABC-P, DBC-P, CSQ, PDDBI-SV, SSP-2	CGI and SRS-2 scores were not significantly different between OXT and placebo groups in the older age group (6–12); there was no significant effect on SRS-2 scores in the younger age group (3–5), but the younger treatment group showed greater improvements in CGI scores than did their older age group counterparts. There were no significant effects of OXT administration on secondary measures.	None reported.
Le, 2022 [75]	41	Autism spectrum disorder (ASD)	Children aged 3–8 with a formal diagnosis of ASD.	Intranasal	24 IU every other day followed by positive social interaction.	6 weeks	ADOS-2, SRS-2; secondary measures included RBS-R, ABAS-II, SCQ, CSQ and eye tracking.	Significant improvement in ADOS-2 and SRS-2 in the treatment group relative to placebo. Greater improvement over placebo was also observed in the OXT group’s ABAS-II and RBS-R scores. Eye tracking data revealed increased time spent viewing dynamic social stimuli versus geometric stimuli.	None reported.
Takiguchi, 2023 [77]	24	Reactive attachment disorder (RAD)	Adolescent males aged 10–18 diagnosed with RAD.	Intranasal	24 IU or Placebo	Variable; mean number of days between scans = 36.3 ± 37.1; TD group, 40.8 ± 28.2; RAD group, 31.2 ± 44.5; *p* = 0.366	fMRI, subjective motivation on visual analog scale.	Enhanced activation of the right middle frontal gyrus and striatum (consistent with the enhancements in the mesolimbic dopaminergic reward system), reduced activation in the right precentral gyrus during reward task. Single dose of intranasal OXT improved motivation and increased neural reward system activity.	None reported.
Karbasi, 2023 [76]	70	Autism spectrum disorder (ASD)	Children aged 4–17 with a formal diagnosis of ASD.	Intranasal	24 IU followed by an ABA session 15 minutes later once per week	6 weeks	SDQ, GARS-2	Significant improvement to GARS-2 and teacher and parent SDQ scores compared to placebo.	None reported.

## Data Availability

Public domain data is available by contacting the corresponding author (Dr. Soonjo Hwang).

## References

[1] Gordon I, Jack A, Pretzsch C, Vander Wyk B, Leckman JF, Feldman R, Intranasal Oxytocin Enhances Connectivity in the Neural Circuitry Supporting Social Motivation and Social Perception in Children with Autism. Sci Rep. 2016 Nov 15;6:35054. doi: 10.1038/srep35054 27845765 PMC5109935

[2] Froemke RC, Young LJ. Oxytocin, Neural Plasticity, and Social Behavior. Annu Rev Neurosci. 2021 Jul 8;44:359–81. doi: 10.1146/annurev-neuro-102320-102847 33823654 PMC8604207

[3] Adolphs R The social brain: neural basis of social knowledge. Annu Rev Psychol. 2009;60:693–716. doi: 10.1146/annurev.psych.60.110707.163514 18771388 PMC2588649

[4] Gimpl G, Fahrenholz F. The oxytocin receptor system: structure, function, and regulation. Physiol Rev. 2001 Apr;81(2):629–83. doi: 10.1152/physrev.2001.81.2.629 11274341

[5] Veening JG, Olivier B. Intranasal administration of oxytocin: behavioral and clinical effects, a review. Neurosci Biobehav Rev. 2013 Sep;37(8):1445–65. doi: 10.1016/j.neubiorev.2013.04.012 23648680 PMC7112651

[6] Kirsch P, Esslinger C, Chen Q, Mier D, Lis S, Siddhanti S, Oxytocin modulates neural circuitry for social cognition and fear in humans. J Neurosci. 2005 Dec 7;25(49):11489–93. doi: 10.1523/JNEUROSCI.3984-05.2005 16339042 PMC6725903

[7] Grace SA, Rossell SL, Heinrichs M, Kordsachia C, Labuschagne I. Oxytocin and brain activity in humans: A systematic review and coordinate-based meta-analysis of functional MRI studies. Psychoneuroendocrinology. 2018 Oct;96:6–24. doi: 10.1016/j.psyneuen.2018.05.031 29879563

[8] Gordon I, Vander Wyk BC, Bennett RH, Cordeaux C, Lucas MV, Eilbott JA, -. Oxytocin enhances brain function in children with autism. Proc Natl Acad Sci U S A. 2013 Dec 24;110(52):20953–8. doi: 10.1073/pnas.1312857110 24297883 PMC3876263

[9] Domes G, Heinrichs M, Kumbier E, Grossmann A, Hauenstein K, Herpertz SC. Effects of intranasal oxytocin on the neural basis of face processing in autism spectrum disorder. Biol Psychiatry. 2013 Aug 1;74(3):164–71. doi: 10.1016/j.biopsych.2013.02.007 23510581

[10] Gamer M, Zurowski B, Büchel C. Different amygdala subregions mediate valence-related and attentional effects of oxytocin in humans. Proc Natl Acad Sci U S A. 2010 May 18;107(20):9400–5. doi: 10.1073/pnas.1000985107 20421469 PMC2889107

[11] Watanabe T, Kuroda M, Kuwabara H, Aoki Y, Iwashiro N, Tatsunobu N, Clinical and neural effects of six-week administration of oxytocin on core symptoms of autism. Brain. 2015 Nov;138(Pt 11):3400–12. doi: 10.1093/brain/awv249 26336909

[12] Kendrick KM, Guastella AJ, Becker B. Overview of Human Oxytocin Research. Curr Top Behav Neurosci. 2018;35:321–348. doi: 10.1007/7854_2017_19 28864976

[13] Oxytocin. Available from: https://pubchem.ncbi.nlm.nih.gov/compound/Oxytocin. Accessed 2023 August 31.

[14] Dopamine. Available from: https://pubchem.ncbi.nlm.nih.gov/compound/Dopamine. Accessed 2023 August 31.

[15] Buijs RM. Intra- and extrahypothalamic vasopressin and oxytocin pathways in the rat. Pathways to the limbic system, medulla oblongata and spinal cord. Cell Tissue Res. 1978 Sep 26;192(3):423–35. doi: 10.1007/BF00212323 699026

[16] Descamps D, Evnouchidou I, Caillens V, Drajac C, Riffault S, van Endert P, The Role of Insulin Regulated Aminopeptidase in Endocytic Trafficking and Receptor Signaling in Immune Cells. Front Mol Biosci. 2020 Oct 20;7:583556. doi: 10.3389/fmolb.2020.583556 33195428 PMC7606930

[17] Georgiadis D, Ziotopoulou A, Kaloumenou E, Lelis A, Papasava A. The Discovery of Insulin-Regulated Aminopeptidase (IRAP) Inhibitors: A Literature Review. Front Pharmacol. 2020 Sep 23;11:585838. doi: 10.3389/fphar.2020.585838 33071797 PMC7538644

[18] Gossen A, Hahn A, Westphal L, Prinz S, Schultz RT, Gründer G, Oxytocin plasma concentrations after single intranasal oxytocin administration—a study in healthy men. Neuropeptides. 2012 Oct;46(5):211–5. doi: 10.1016/j.npep.2012.07.001 22884888

[19] Feifel D, Shilling PD, MacDonald K. A Review of Oxytocin’s Effects on the Positive, Negative, and Cognitive Domains of Schizophrenia. Biol Psychiatry. 2016 Feb 1;79(3):222–33. doi: 10.1016/j.biopsych.2015.07.025 26410353 PMC5673255

[20] Herpertz S, Nizielski S, Hock M, Schütz A. The Relevance of Emotional Intelligence in Personnel Selection for High Emotional Labor Jobs. PLoS One. 2016 Apr 28;11(4):e0154432. doi: 10.1371/journal.pone.0154432 27124201 PMC4849674

[21] Domes G, Ower N, von Dawans B, Spengler FB, Dziobek I, Bohus M, Effects of intranasal oxytocin administration on empathy and approach motivation in women with borderline personality disorder: a randomized controlled trial. Transl Psychiatry. 2019 Dec 4;9(1):328. doi: 10.1038/s41398-019-0658-4 31801937 PMC6892895

[22] Munesue T, Nakamura H, Kikuchi M, Miura Y, Takeuchi N, Anme T, Oxytocin for Male Subjects with Autism Spectrum Disorder and Comorbid Intellectual Disabilities: A Randomized Pilot Study. Front Psychiatry. 2016 Jan 21;7:2. doi: 10.3389/fpsyt.2016.00002 26834651 PMC4720778

[23] Scantamburlo G, Hansenne M, Geenen V, Legros JJ, Ansseau M. Additional intranasal oxytocin to escitalopram improves depressive symptoms in resistant depression: an open trial. Eur Psychiatry. 2015 Jan;30(1):65–8. doi: 10.1016/j.eurpsy.2014.08.007 25282363

[24] Corbett BA, Bales KL, Swain D, Sanders K, Weinstein TA, Muglia LJ. Comparing oxytocin and cortisol regulation in a double-blind, placebo-controlled, hydrocortisone challenge pilot study in children with autism and typical development. J Neurodev Disord. 2016 Aug 18;8:32. doi: 10.1186/s11689-016-9165-6 27540420 PMC4989357

[25] Dadds MR, MacDonald E, Cauchi A, Williams K, Levy F, Brennan J. Nasal oxytocin for social deficits in childhood autism: a randomized controlled trial. J Autism Dev Disord. 2014 Mar;44(3):521–31. doi: 10.1007/s10803-013-1899-3 23888359

[26] Sikich L, Kolevzon A, King BH, McDougle CJ, Sanders KB, Kim SJ, Intranasal Oxytocin in Children and Adolescents with Autism Spectrum Disorder. N Engl J Med. 2021 Oct 14;385(16):1462–73. doi: 10.1056/NEJMoa2103583 34644471 PMC9701092

[27] Bigler ED, Mortensen S, Neeley ES, Ozonoff S, Krasny L, Johnson M, Superior temporal gyrus, language function, and autism. Dev Neuropsychol. 2007;31(2):217–38. doi: 10.1080/87565640701190841 17488217

[28] Oiesvold T, Nivison M, Hansen V, Skre I, Ostensen L, Sørgaard KW. Diagnosing comorbidity in psychiatric hospital: challenging the validity of administrative registers. BMC Psychiatry. 2013 Jan 8;13:13. doi: 10.1186/1471-244X-13-13 23297686 PMC3544620

[29] Dell’osso L, Pini S. What Did We Learn from Research on Comorbidity In Psychiatry? Advantages and Limitations in the Forthcoming DSM-V Era. Clin Pract Epidemiol Ment Health. 2012;8:180–4. doi: 10.2174/1745017901208010180 23304235 PMC3537081

[30] Banerjee A, Chitnis UB, Jadhav SL, Bhawalkar JS, Chaudhury S. Hypothesis testing, type I and type II errors. Ind Psychiatry J. 2009 Jul;18(2):127–31. doi: 10.4103/0972-6748.62274 21180491 PMC2996198

[31] Leng G, Ludwig M. Intranasal Oxytocin: Myths and Delusions. Biol Psychiatry. 2016 Feb 1;79(3):243–50. doi: 10.1016/j.biopsych.2015.05.003 26049207

[32] Leibenluft E Pediatric Irritability: A Systems Neuroscience Approach. Trends Cogn Sci. 2017 Apr;21(4):277–89. doi: 10.1016/j.tics.2017.02.002 28274677 PMC5366079

[33] Leibenluft E, Kircanski K. Chronic Irritability in Youth: A Reprise on Challenges and Opportunities Toward Meeting Unmet Clinical Needs. Child Adolesc Psychiatr Clin N Am. 2021 Jul;30(3):667–83. doi: 10.1016/j.chc.2021.04.014 34053693 PMC13317484

[34] Leibenluft E, Stoddard J. The developmental psychopathology of irritability. Dev Psychopathol. 2013 Nov;25(4 Pt 2):1473–87. doi: 10.1017/S0954579413000722 24342851 PMC4476313

[35] Kempes M, Matthys W, de Vries H, van Engeland H. Reactive and proactive aggression in children--a review of theory, findings and the relevance for child and adolescent psychiatry. Eur Child Adolesc Psychiatry. 2005 Feb;14(1):11–9. doi: 10.1007/s00787-005-0432-4 15756511

[36] Radke S, Volman I, Kokal I, Roelofs K, de Bruijn ERA, Toni I. Oxytocin reduces amygdala responses during threat approach. Psychoneuroendocrinology. 2017 May;79:160–6. doi: 10.1016/j.psyneuen.2017.02.028 28285187

[37] Kanat M, Heinrichs M, Schwarzwald R, Domes G. Oxytocin attenuates neural reactivity to masked threat cues from the eyes. Neuropsychopharmacology. 2015 Jan;40(2):287–95. doi: 10.1038/npp.2014.183 25047745 PMC4443952

[38] Bertsch K, Gamer M, Schmidt B, Schmidinger I, Walther S, Kästel T, Oxytocin and reduction of social threat hypersensitivity in women with borderline personality disorder. Am J Psychiatry. 2013 Oct;170(10):1169–77. doi: 10.1176/appi.ajp.2013.13020263 23982273

[39] Insel TR. Translating Oxytocin Neuroscience to the Clinic: A National Institute of Mental Health Perspective. Biol Psychiatry. 2016 Feb 1;79(3):153–4. doi: 10.1016/j.biopsych.2015.02.002 26723108

[40] Crum KI, Hwang S, Blair KS, Aloi JM, Meffert H, White SF, Interaction of irritability and anxiety on emotional responding and emotion regulation: a functional MRI study. Psychol Med. 2021 Dec;51(16):2778–88. doi: 10.1017/S0033291720001397 32584213 PMC7759590

[41] Mukherjee P, Vilgis V, Rhoads S, Chahal R, Fassbender C, Leibenluft E, Associations of Irritability With Functional Connectivity of Amygdala and Nucleus Accumbens in Adolescents and Young Adults With ADHD. J Atten Disord. 2022 May;26(7):1040–1050. doi: 10.1177/10870547211057074 34724835 PMC8957582

[42] Damen L, Grootjen LN, Juriaans AF, Donze SH, Huisman TM, Visser JA, Oxytocin in young children with Prader-Willi syndrome: Results of a randomized, double-blind, placebo-controlled, crossover trial investigating 3 months of oxytocin. Clin Endocrinol (Oxf). 2021 May;94(5):774–85. doi: 10.1111/cen.14387 33296519 PMC8246775

[43] Miller JL, Tamura R, Butler MG, Kimonis V, Sulsona C, Gold JA, Oxytocin treatment in children with Prader-Willi syndrome: A double-blind, placebo-controlled, crossover study. Am J Med Genet A. 2017 May;173(5):1243–50. doi: 10.1002/ajmg.a.38160 28371242 PMC5828021

[44] Fastman J, Foss-Feig J, Frank Y, Halpern D, Harony-Nicolas H, Layton C, A randomized controlled trial of intranasal oxytocin in Phelan-McDermid syndrome. Mol Autism. 2021 Sep 30;12(1):62. doi: 10.1186/s13229-021-00459-1 34593045 PMC8482590

[45] Kalvin CB, Gladstone TR, Jordan R, Rowley S, Marsh CL, Ibrahim K, Assessing Irritability in Children with Autism Spectrum Disorder Using the Affective Reactivity Index. J Autism Dev Disord. 2021 May;51(5):1496–1507. doi: 10.1007/s10803-020-04627-9 32734421 PMC7855357

[46] Schwartz L, Caixàs A, Dimitropoulos A, Dykens E, Duis J, Einfeld S, Gallagher L, Holland A, Rice L, Roof E, Salehi P, Strong T, Taylor B, Woodcock K. Behavioral features in Prader-Willi syndrome (PWS): consensus paper from the International PWS Clinical Trial Consortium. J Neurodev Disord. 2021 Jun 21;13(1):25. doi: 10.1186/s11689-021-09373-2 34148559 PMC8215770

[47] Kohlenberg TM, Trelles MP, McLarney B, Betancur C, Thurm A, Kolevzon A. Psychiatric illness and regression in individuals with Phelan-McDermid syndrome. J Neurodev Disord. 2020 Feb 12;12(1):7. doi: 10.1186/s11689-020-9309-6 32050889 PMC7014655

[48] Bos PA, Panksepp J, Bluthé RM, van Honk J. Acute effects of steroid hormones and neuropeptides on human social-emotional behavior: a review of single administration studies. Front Neuroendocrinol. 2012 Jan;33(1):17–35. doi: 10.1016/j.yfrne.2011.01.002 21256859

[49] Parker KJ, Garner JP, Libove RA, Hyde SA, Hornbeak KB, Carson DS, Plasma oxytocin concentrations and OXTR polymorphisms predict social impairments in children with and without autism spectrum disorder. Proc Natl Acad Sci U S A. 2014 Aug 19;111(33):12258–63. doi: 10.1073/pnas.1402236111 25092315 PMC4143031

[50] LoParo D, Waldman ID. The oxytocin receptor gene (OXTR) is associated with autism spectrum disorder: a meta-analysis. Mol Psychiatry. 2015 May;20(5):640–6. doi: 10.1038/mp.2014.77 25092245

[51] Al-Ali Z, Yasseen AA, Al-Dujailli A, Al-Karaqully AJ, McAllister KA, Jumaah AS. The oxytocin receptor gene polymorphism rs2268491 and serum oxytocin alterations are indicative of autism spectrum disorder: A case-control paediatric study in Iraq with personalized medicine implications. PLoS One. 2022 Mar 22;17(3):e0265217. doi: 10.1371/journal.pone.0265217 35316293 PMC8939799

[52] Mayes SD, Waxmonsky J, Calhoun SL, Kokotovich C, Mathiowetz C, Baweja R. Disruptive mood dysregulation disorder (DMDD) symptoms in children with autism, ADHD, and neurotypical development and impact of co-occurring ODD, depression, and anxiety. Res Autism Spec Disord. 2015 Oct;18:64–72. doi: 10.1016/j.rasd.2015.07.003

[53] Mikita N, Hollocks MJ, Papadopoulos AS, Aslani A, Harrison S, Leibenluft E, Irritability in boys with autism spectrum disorders: an investigation of physiological reactivity. J Child Psychol Psychiatry. 2015 Oct;56(10):1118–26. doi: 10.1111/jcpp.12382 25626926 PMC4737220

[54] Bennett JA, Germani T, Haqq AM, Zwaigenbaum L. Autism spectrum disorder in Prader-Willi syndrome: A systematic review. Am J Med Genet A. 2015 Dec;167A(12):2936–44. doi: 10.1002/ajmg.a.37286 26331980

[55] McCandless SE; Committee on Genetics. Clinical report—health supervision for children with Prader-Willi syndrome. Pediatrics. 2011 Jan;127(1):195–204. doi: 10.1542/peds.2010-2820 21187304

[56] Rice LJ, Agu J, Carter CS, Harris JC, Nazarloo HP, Naanai H, The relationship between endogenous oxytocin and vasopressin levels and the Prader-Willi syndrome behaviour phenotype. Front Endocrinol. 2023 May 29;14:1183525. doi: 10.3389/fendo.2023.1183525 PMC10259653 37313445

[57] Oztan O, Zyga O, Stafford DEJ, Parker KJ. Linking oxytocin and arginine vasopressin signaling abnormalities to social behavior impairments in Prader-Willi syndrome. Neurosci Biobehav Rev. 2022 Nov;142:104870. doi: 10.1016/j.neubiorev.2022.104870 36113782 PMC11024898

[58] Phelan MC. Deletion 22q13.3 syndrome. Orphanet J Rare Dis. 2008 May 27;3:14. doi: 10.1186/1750-1172-3-14. 18505557 PMC2427010

[59] Moessner R, Marshall CR, Sutcliffe JS, Skaug J, Pinto D, Vincent J, Contribution of SHANK3 mutations to autism spectrum disorder. Am J Hum Genet. 2007 Dec;81(6):1289–97. doi: 10.1086/522590 17999366 PMC2276348

[60] Harony-Nicolas H, Kay M, du Hoffmann J, Klein ME, Bozdagi-Gunal O, Riad M, Oxytocin improves behavioral and electrophysiological deficits in a novel Shank3-deficient rat. Elife. 2017 Jan 31;6:e18904. doi: 10.7554/eLife.18904 28139198 PMC5283828

[61] Ellis EE, Yilanli M, Saadabadi A. Reactive Attachment Disorder. Treasure Island (FL, US): StatPearls Publishing; 2023.

[62] Sterne JAC, Savović J, Page MJ, Elbers RG, Blencowe NS, Boutron I, RoB 2: a revised tool for assessing risk of bias in randomised trials. BMJ. 2019 Aug 28;366:l4898. doi: 10.1136/bmj.l4898 31462531

[63] Schmidt JD, Huete JM, Fodstad JC, Chin MD, Kurtz PF. An evaluation of the Aberrant Behavior Checklist for children under age 5. Res Dev Disabil. 2013 Apr;34(4):1190–7. doi: 10.1016/j.ridd.2013.01.002 23376629 PMC3632068

[64] Bishop SL, Hus V, Duncan A, Huerta M, Gotham K, Pickles A, Subcategories of restricted and repetitive behaviors in children with autism spectrum disorders. J Autism Dev Disord. 2013 Jun;43(6):1287–97. doi: 10.1007/s10803-012-1671-0 23065116 PMC3579001

[65] Kerr-Gaffney J, Harrison A, Tchanturia K. The social responsiveness scale is an efficient screening tool for autism spectrum disorder traits in adults with anorexia nervosa. Eur Eat Disord Rev. 2020 Jul;28(4):433–44. doi: 10.1002/erv.2736 32243021 PMC8653883

[66] Marzocchi GM, Capron C, Di Pietro M, Duran Tauleria E, Duyme M, Frigerio A, The use of the Strengths and Difficulties Questionnaire (SDQ) in Southern European countries. Eur Child Adolesc Psychiatry. 2004;13 Suppl 2:II40–6. doi: 10.1007/s00787-004-2007-1 15243785

[67] Stringaris A, Goodman R, Ferdinando S, Razdan V, Muhrer E, Leibenluft E, The Affective Reactivity Index: a concise irritability scale for clinical and research settings. J Child Psychol Psychiatry. 2012 Nov;53(11):1109–17. doi: 10.1111/j.1469-7610.2012.02561.x 22574736 PMC3484687

[68] Saatchi B, Agbayani CG, Clancy SL, Fortier MA. Measuring irritability in young adults: An integrative review of measures and their psychometric properties. J Psychiatr Ment Health Nurs. 2023 Feb;30(1):35–53. doi: 10.1111/jpm.12851 35716348 PMC10084147

[69] Greene RK, Spanos M, Alderman C, Walsh E, Bizzell J, Mosner MG, The effects of intranasal oxytocin on reward circuitry responses in children with autism spectrum disorder. J Neurodev Disord. 2018 Mar 27;10(1):12. doi: 10.1186/s11689-018-9228-y 29587625 PMC5870086

[70] Yatawara CJ, Einfeld SL, Hickie IB, Davenport TA, Guastella AJ. The effect of oxytocin nasal spray on social interaction deficits observed in young children with autism: a randomized clinical crossover trial. Mol Psychiatry. 2016 Sep;21(9):1225–31. doi: 10.1038/mp.2015.162 26503762 PMC4995545

[71] Parker KJ, Oztan O, Libove RA, Sumiyoshi RD, Jackson LP, Karhson DS, Intranasal oxytocin treatment for social deficits and biomarkers of response in children with autism. Proc Natl Acad Sci U S A. 2017 Jul 25;114(30):8119–24. doi: 10.1073/pnas.1705521114 28696286 PMC5544319

[72] Daniels N, Moerkerke M, Steyaert J, Bamps A, Debbaut E, Prinsen J, Effects of multiple-dose intranasal oxytocin administration on social responsiveness in children with autism: a randomized, placebo-controlled trial. Mol Autism. 2023 Apr 20;14(1):16. doi: 10.1186/s13229-023-00546-5 PMC10117268 37081454

[73] Korisky A, Goldstein A, Gordon I. The dual neural effects of oxytocin in autistic youth: results from a randomized trial. Sci Rep. 2022 Sep 29;12(1):16304. doi: 10.1038/s41598-022-19524-7 36175473 PMC9523043

[74] Guastella AJ, Boulton KA, Whitehouse AJO, Song YJ, Thapa R, Gregory SG, The effect of oxytocin nasal spray on social interaction in young children with autism: a randomized clinical trial. Mol Psychiatry. 2023 Feb;28(2):834–42. doi: 10.1038/s41380-022-01845-8 36302965 PMC9607840

[75] Le J, Zhang L, Zhao W, Zhu S, Lan C, Kou J, Infrequent Intranasal Oxytocin Followed by Positive Social Interaction Improves Symptoms in Autistic Children: A Pilot Randomized Clinical Trial. Psychother Psychosom. 2022;91(5):335–47. doi: 10.1159/000524543 35545057

[76] Karbasi A, Shafiezadegan Isfahani S, Maracy MR, Sabzghabaee AM. Effect of intranasal oxytocin combination therapy with applied behavior analysis on social impairments in pediatric’s children with autism spectrum disorder. Middle East Curr Psychiatry. 2023;30:35. doi: 10.1186/s43045-023-00300-w

[77] Takiguchi S, Makita K, Fujisawa TX, Nishitani S, Tomoda A. Effects of intranasal oxytocin on neural reward processing in children and adolescents with reactive attachment disorder: A randomized controlled trial. Front. Child Adolesc. Psychiatry. 2022;1. doi: 10.3389/frcha.2022.1056115 PMC11748893 39839202

[78] O’Connell LA, Hofmann HA. The vertebrate mesolimbic reward system and social behavior network: a comparative synthesis. J Comp Neurol. 2011 Dec 15;519(18):3599–639. doi: 10.1002/cne.22735 21800319

[79] Roesch MR, Singh T, Brown PL, Mullins SE, Schoenbaum G. Ventral striatal neurons encode the value of the chosen action in rats deciding between differently delayed or sized rewards. J Neurosci. 2009 Oct 21;29(42):13365–76. doi: 10.1523/JNEUROSCI.2572-09.2009 19846724 PMC2788608

[80] Apps MA, Rushworth MF, Chang SW. The Anterior Cingulate Gyrus and Social Cognition: Tracking the Motivation of Others. Neuron. 2016 May 18;90(4):692–707. doi: 10.1016/j.neuron.2016.04.018 27196973 PMC4885021

[81] Hertrich I, Dietrich S, Blum C, Ackermann H. The Role of the Dorsolateral Prefrontal Cortex for Speech and Language Processing. Front Hum Neurosci. 2021 May 17;15:645209. doi: 10.3389/fnhum.2021.645209 34079444 PMC8165195

[82] Cai RY, Richdale AL, Uljarević M, Dissanayake C, Samson AC. Emotion regulation in autism spectrum disorder: Where we are and where we need to go. Autism Res. 2018 Jul;11(7):962–78. doi: 10.1002/aur.1968 29979494

[83] Keluskar J, Reicher D, Gorecki A, Mazefsky C, Crowell JA. Understanding, Assessing, and Intervening with Emotion Dysregulation in Autism Spectrum Disorder: A Developmental Perspective. Child Adolesc Psychiatr Clin N Am. 2021 Apr;30(2):335–48. doi: 10.1016/j.chc.2020.10.013 33743942

[84] Neumann ID, Maloumby R, Beiderbeck DI, Lukas M, Landgraf R. Increased brain and plasma oxytocin after nasal and peripheral administration in rats and mice. Psychoneuroendocrinology. 2013 Oct;38(10):1985–93. doi: 10.1016/j.psyneuen.2013.03.003 23579082

[85] Bauman MD, Murai T, Hogrefe CE, Platt ML. Opportunities and challenges for intranasal oxytocin treatment studies in nonhuman primates. Am J Primatol. 2018 Oct;80(10):e22913. doi: 10.1002/ajp.22913 30281820 PMC6690341

[86] Freeman SM, Samineni S, Allen PC, Stockinger D, Bales KL, Hwa GG, Plasma and CSF oxytocin levels after intranasal and intravenous oxytocin in awake macaques. Psychoneuroendocrinology. 2016 Apr;66:185–94. doi: 10.1016/j.psyneuen.2016.01.014 26826355

[87] Lee MR, Shnitko TA, Blue SW, Kaucher AV, Winchell AJ, Erikson DW, Labeled oxytocin administered via the intranasal route reaches the brain in rhesus macaques. Nat Commun. 2020 Jun 3;11(1):2783. doi: 10.1038/s41467-020-15942-1 32494001 PMC7270110

[88] Striepens N, Kendrick KM, Hanking V, Landgraf R, Wüllner U, Maier W, Elevated cerebrospinal fluid and blood concentrations of oxytocin following its intranasal administration in humans. Sci Rep. 2013 Dec 6;3:3440. doi: 10.1038/srep03440 24310737 PMC3853684

[89] Quintana DS, Lischke A, Grace S, Scheele D, Ma Y, Becker B. Advances in the field of intranasal oxytocin research: lessons learned and future directions for clinical research. Mol Psychiatry. 2021 Jan;26(1):80–91. doi: 10.1038/s41380-020-00864-7 32807845 PMC7815514

[90] Mens WB, Witter A, van Wimersma Greidanus TB. Penetration of neurohypophyseal hormones from plasma into cerebrospinal fluid (CSF): half-times of disappearance of these neuropeptides from CSF. Brain Res. 1983 Feb 28;262(1):143–9. doi: 10.1016/0006-8993(83)90478-x 6831225

[91] Quintana DS, Westlye LT, Rustan ØG, Tesli N, Poppy CL, Smevik H, Low-dose oxytocin delivered intranasally with Breath Powered device affects social-cognitive behavior: a randomized four-way crossover trial with nasal cavity dimension assessment. Transl Psychiatry. 2015 Jul 14;5(7):e602. doi: 10.1038/tp.2015.93 26171983 PMC5068727

[92] Quintana DS, Westlye LT, Alnæs D, Rustan ØG, Kaufmann T, Smerud KT, Low dose intranasal oxytocin delivered with Breath Powered device dampens amygdala response to emotional stimuli: A peripheral effect-controlled within-subjects randomized dose-response fMRI trial. Psychoneuroendocrinology. 2016 Jul;69:180–8. doi: 10.1016/j.psyneuen.2016.04.010 27107209

[93] Quintana DS, Westlye LT, Alnæs D, Kaufmann T, Mahmoud RA, Smerud KT, Low-dose intranasal oxytocin delivered with Breath Powered device modulates pupil diameter and amygdala activity: a randomized controlled pupillometry and fMRI study. Neuropsychopharmacology. 2019 Jan;44(2):306–13. doi: 10.1038/s41386-018-0241-3 30323359 PMC6300535

[94] Gouveia FV, Hamani C, Fonoff ET, Brentani H, Alho EJL, de Morais RMCB, Amygdala and Hypothalamus: Historical Overview With Focus on Aggression. Neurosurgery. 2019 Jul 1;85(1):11–30. doi: 10.1093/neuros/nyy635 30690521 PMC6565484

[95] Berends YR, Tulen JHM, Wierdsma AI, van Pelt J, Kushner SA, van Marle HJC. Oxytocin, vasopressin and trust: Associations with aggressive behavior in healthy young males. Physiol Behav. 2019 May 15;204:180–5. doi: 10.1016/j.physbeh.2019.02.027 30802507

[96] Berends YR, Tulen JHM, Wierdsma AI, van Pelt J, Feldman R, Zagoory-Sharon O, Intranasal administration of oxytocin decreases task-related aggressive responses in healthy young males. Psychoneuroendocrinology. 2019 Aug;106:147–54. doi: 10.1016/j.psyneuen.2019.03.027 30981088

[97] Alcorn JL III, Green CE, Schmitz J, Lane SD. Effects of oxytocin on aggressive responding in healthy adult men. Behav Pharmacol. 2015 Dec;26(8 Spec No):798–804. doi: 10.1097/FBP.0000000000000173 26241153 PMC4631637

[98] Ne’eman R, Perach-Barzilay N, Fischer-Shofty M, Atias A, Shamay-Tsoory SG. Intranasal administration of oxytocin increases human aggressive behavior. Horm Behav. 2016 Apr;80:125–31. doi: 10.1016/j.yhbeh.2016.01.015 26862988

[99] Koch SB, van Zuiden M, Nawijn L, Frijling JL, Veltman DJ, Olff M. Intranasal Oxytocin Administration Dampens Amygdala Reactivity towards Emotional Faces in Male and Female PTSD Patients. Neuropsychopharmacology. 2016 May;41(6):1495–504. doi: 10.1038/npp.2015.299 26404844 PMC4832009

[100] Labuschagne I, Phan KL, Wood A, Angstadt M, Chua P, Heinrichs M, Oxytocin attenuates amygdala reactivity to fear in generalized social anxiety disorder. Neuropsychopharmacology. 2010 Nov;35(12):2403–13. doi: 10.1038/npp.2010.123 20720535 PMC3055328

[101] Lischke A, Herpertz SC, Berger C, Domes G, Gamer M. Divergent effects of oxytocin on (para-)limbic reactivity to emotional and neutral scenes in females with and without borderline personality disorder. Soc Cogn Affect Neurosci. 2017 Nov 1;12(11):1783–92. doi: 10.1093/scan/nsx107 29036358 PMC5714167

[102] Spengler FB, Schultz J, Scheele D, Essel M, Maier W, Heinrichs M, Kinetics and Dose Dependency of Intranasal Oxytocin Effects on Amygdala Reactivity. Biol Psychiatry. 2017 Dec 15;82(12):885–94. doi: 10.1016/j.biopsych.2017.04.015 28629540

